# Open-Set Recognition of Individual Cows Based on Spatial Feature Transformation and Metric Learning

**DOI:** 10.3390/ani14081175

**Published:** 2024-04-14

**Authors:** Buyu Wang, Xia Li, Xiaoping An, Weijun Duan, Yuan Wang, Dian Wang, Jingwei Qi

**Affiliations:** 1 College of Computer and Information Engineering, Inner Mongolia Agricultural University, Hohhot 010010, China; bywang08@imau.edu.cn (B.W.); 2020302100004@emails.imau.edu.cn (W.D.); 2 Key Laboratory of Smart Animal Husbandry at Universities of Inner Mongolia Autonomous Region, Inner Mongolia Agricultural University, Hohhot 010010, China; anxiaoping@imau.edu.cn (X.A.); wangyuan@imau.edu.cn (Y.W.); 3 National Center of Technology Innovation for Dairy-Breeding and Production Research Subcenter, Hohhot 010018, China; wd@yourandairy.com; 4 College of Animal Science, Inner Mongolia Agricultural University, Hohhot 010010, China; lixia2270@emails.imau.edu.cn

**Keywords:** cow individual recognition, open-set recognition, overhead perspective, distance metrics, STN

## Abstract

**Simple Summary:**

Accurate individual cow identification is key to enhancing farm management efficiency. The mainstream method of identifying cows through physical tags is somewhat invasive. This study introduces a non-invasive approach that utilizes overhead cameras to recognize cows without any physical contact. This method adapts well to changes in cow positions and partial obstructions and does not require system adjustments when new cows are introduced. By focusing on unique features in overhead images, this approach facilitates more convenient and effective monitoring and management of cows, assisting farm operators in achieving efficient daily farm operations.

**Abstract:**

The automated recognition of individual cows is foundational for implementing intelligent farming. Traditional methods of individual cow recognition from an overhead perspective primarily rely on singular back features and perform poorly for cows with diverse orientation distributions and partial body visibility in the frame. This study proposes an open-set method for individual cow recognition based on spatial feature transformation and metric learning to address these issues. Initially, a spatial transformation deep feature extraction module, ResSTN, which incorporates preprocessing techniques, was designed to effectively address the low recognition rate caused by the diverse orientation distribution of individual cows. Subsequently, by constructing an open-set recognition framework that integrates three attention mechanisms, four loss functions, and four distance metric methods and exploring the impact of each component on recognition performance, this study achieves refined and optimized model configurations. Lastly, introducing moderate cropping and random occlusion strategies during the data-loading phase enhances the model’s ability to recognize partially visible individuals. The method proposed in this study achieves a recognition accuracy of 94.58% in open-set scenarios for individual cows in overhead images, with an average accuracy improvement of 2.98 percentage points for cows with diverse orientation distributions, and also demonstrates an improved recognition performance for partially visible and randomly occluded individual cows. This validates the effectiveness of the proposed method in open-set recognition, showing significant potential for application in precision cattle farming management.

## 1. Introduction

In the modernization of the livestock industry, precise identification and monitoring of dairy cows have become key to improving production efficiency [1]. Although traditional methods for dairy cow identification, such as ear tags or RFID, are widely used in practice [2,3], they often involve invasive procedures, high maintenance costs, and susceptibility to damage. These issues not only affect the efficiency of farm management but could also potentially impact the welfare of dairy cows. With advances in technology and increased awareness of animal welfare, developing an efficient, non-invasive method of individual identification has become particularly urgent. Currently, research based on deep learning and machine vision has been widely applied across various industries [4,5,6,7], and vision-based dairy cow identification offers a new solution to this problem with its non-invasiveness, low cost, and high efficiency [8].

Holstein cows, as one of the most common and productive dairy breeds globally, have unique black and white patterns that naturally benefit visual-based individual identification [9,10,11]. Zhao et al. (2019) proposed a visual-system-based method for individual dairy cow identification as a potential alternative to RFID. This method uses an adaptive SOM technique to detect the cow’s outline and extract the largest inscribed rectangle to locate the body area. By extracting and matching feature points from the body images, they achieved an accuracy rate of 96.72% [12]. Xiao et al. (2022) explored individual cow identification in an unconstrained barn setting and introduced a method based on an enhanced Mask R-CNN and SVM classifier. They collected overhead images of cows, used the improved Mask R-CNN for image segmentation and the extraction of dorsal shape features, selected the best feature subset with the Fisher method, and employed an SVM classifier for individual identification [13], achieving a 98.67% accuracy rate on a dataset including 48 overhead cow images. Zhang et al. (2023) proposed a new method for dairy cow individual identification based on image binarization and cascaded classification, aimed at addressing the challenges of similar biometric features, unstable image quality in complex environments, and rapid model parameter growth [14]. The study utilized 11,800 side-view walking images of 118 cows to build the dataset, achieving an identification accuracy of 98.5%. In addition to other methods, the facial recognition of cattle remains a significant research focus. Chen et al. (2022) proposed a deep learning model integrating global and local network architectures with attention mechanisms for the enhanced facial recognition of dairy cows [15]. Tests on a dataset containing 130,000 images of 3000 cows showed that this model optimization improved accuracy by 2.8%. Weng et al. (2022) developed a dual-branch convolutional neural network (TB-CNN) model to address variations in posture and shooting angles in cattle facial recognition. This model processes images from different angles using separate CNN channels, enhancing identification accuracy through feature integration and global average pooling [16], achieving a recognition rate exceeding 99.7% across multiple datasets. Other researchers have also proposed various methods for cattle facial recognition [17,18]. Additionally, some studies focus on identifying individuals based on other body parts of cattle [19,20] and on model lightweighting [21,22,23]. However, whether based on the cow’s back, face, or other body parts, most studies on individual dairy cow recognition focus on closed-set identification, which only recognizes individuals within the training set. In real farm production environments, recognition systems need to quickly adapt to the inclusion of new individuals; the limitations of existing research mean that even high accuracy rates may not be applicable in practical production.

Some researchers have attempted open-set identification as a promising application. Andrew et al. (2021) first introduced a complete process for identifying both known and unknown Holstein Friesian cattle. By establishing a robust embedding space based on a few instances, they achieved the efficient identification of unknown cattle, with an average accuracy rate of 93.75% [24]. However, the training sample size was small, and there is still room for accuracy improvement. Wang et al. (2023) introduced the ResNAM network that integrates the Normalized Attention Module (NAM) with the ResNet model. They constructed an open-set facial recognition framework for pigs by incorporating multiple loss functions and metrics, achieving a high accuracy of 95.28% [25]. Meanwhile, Wang et al. (2023) [26] employed the ShuffleNet v2 model combined with triplet loss and cross-entropy loss to enhance the network’s ability to distinguish similar individuals, reaching an open-set recognition accuracy of 82.93% on a dataset of 87 cattle. These studies demonstrate the feasibility of using specific appearances for open-set individual animal recognition in the livestock sector. In practical applications, due to limitations on the placement of visual capture devices and obstructions caused by other animals or environmental structures, the accuracy of recognizing images of faces, sides, and rears often suffers significantly from environmental conditions. Overhead perspective-based individual recognition can better mitigate potential impacts from such conditions.

While mitigating some limitations of traditional methods, existing vision-based identification technologies still show significant shortcomings in addressing challenges such as the diversified orientation distribution of individual cows; partial image visibility; and complex, varying lighting conditions. Additionally, the traditional use of closed-set identification methods can only recognize within a fixed, predefined set of individuals, which clearly cannot meet the practical needs of the livestock industry. These challenges increase the difficulty of identification and limit the widespread application of the technology.

This study addresses the challenges in individual cow recognition by proposing an open-set recognition scheme for individual cows based on spatial feature transformation and metric learning, innovatively incorporating the STN network [27], which enhances recognition performance under diverse orientation distributions of individual cows. It explores the impact of each component on recognition performance by integrating various attention mechanisms, multiple loss functions, and distance metric methods. The introduction of moderate cropping and random occlusion strategies during the data-loading phase significantly improves the model’s ability to recognize partially visible individuals. It assesses the impact of different lighting conditions and enhancement measures on performance. The main contributions of this study are as follows:

(1) By constructing an open-set individual cow recognition framework that combines “spatial feature transformation and metric learning”, this study successfully validates the effectiveness of open-set recognition using complete cow images from an overhead perspective.
(2) A new deep learning model, ResSTN, is designed, which significantly enhances the model’s adaptability to challenges such as diverse orientation distributions, complex lighting conditions, and partial occlusions of individual cows by integrating the STN network and attention mechanisms.
(3) The detailed comparative analysis delves into the application effects of different attention mechanisms, loss functions, and distance metric methods in the open-set recognition of individual cows from an overhead perspective. It provides empirical evidence for understanding the practicality and limitations of various methods in cow individual recognition, laying the foundation for further research on optimizing and improving open-set recognition algorithms in specific application scenarios.
(4) This research offers a high-performance cow monitoring solution for intelligent livestock farming, contributing to enhanced management efficiency in cow production and showcasing the tremendous potential of “spatial feature transformation feature extraction + metric learning” technology in the livestock industry.

## 2. Materials and Methods

### 2.1. Data Collection

#### 2.1.1. Video Data Collection

**Figure 1:**
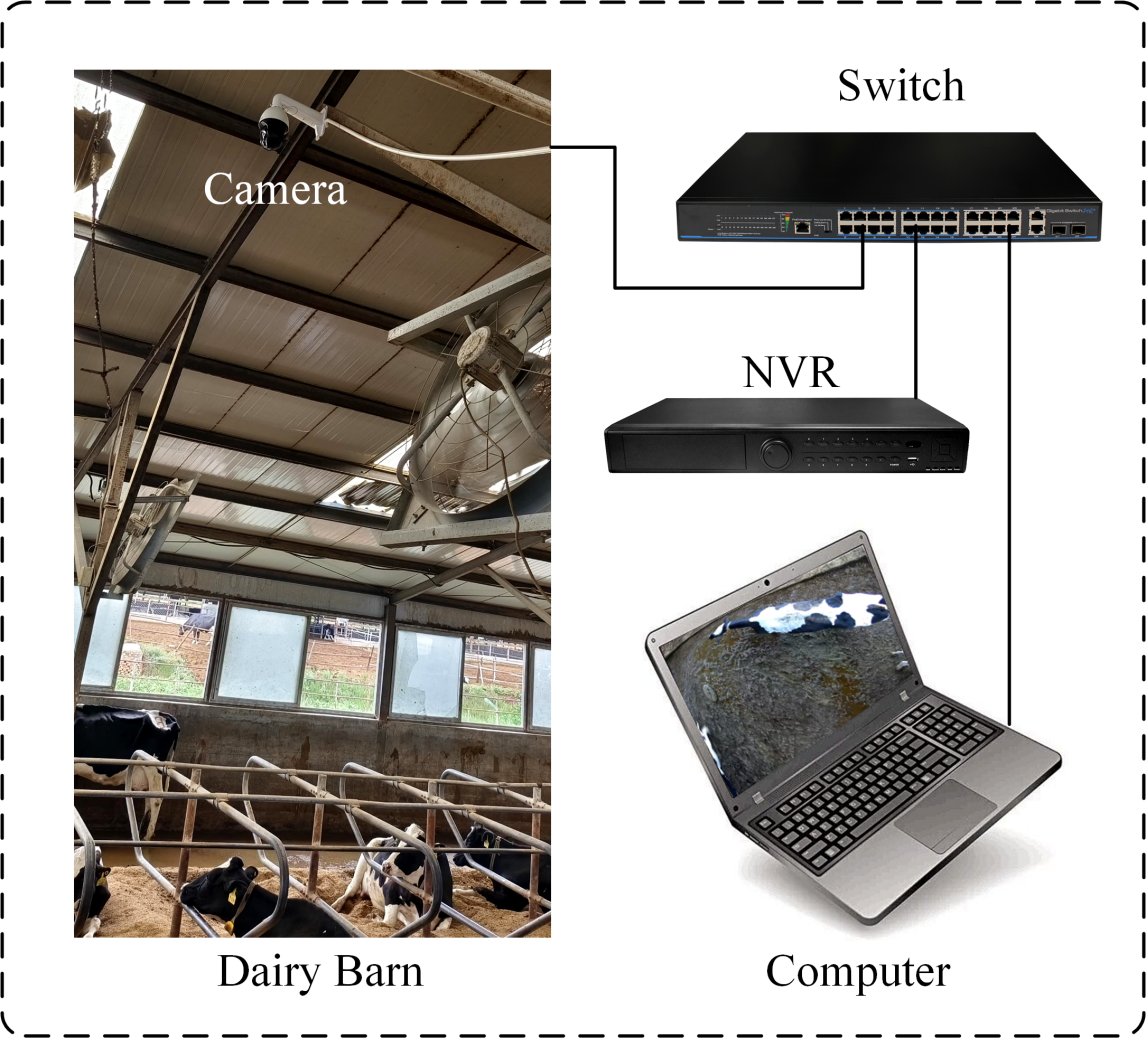
Video data collection.

The data for this study were collected in the summer of 2023 from a large commercial dairy farm in Hohhot, Inner Mongolia, focusing on adult Holstein cows. The data were captured using a DS-2DC4223IW-D spherical camera (Hikvision, Hangzhou, China) with an 8 mm focal length and a 57.6° field of view. It features a pan-tilt head capable of 360° horizontal and −15° to 90° vertical movement, supporting 23× optical zoom and 16× digital zoom, and records at a resolution of 1920 × 1080. The camera was mounted at a height of 4.3 m above the cowshed floor, positioned vertically to film the cows. Data were continuously collected as video streams for 16 days and automatically stored in real-time on the farm’s central NVR, model DS-7932N-R4 (Hikvision, Hangzhou, China). The cowshed was open, with no enclosures except for barrier railings, ensuring good ventilation and lighting conditions. At night, lighting was provided by cowshed lamps, with lights automatically turned off at 22:30 by a timer switch. Video data were accessed via a laptop connected to the NVR on the farm’s local network, selecting 327 usable video files in H.265 format with .mp4 extensions. The video data collection scheme is shown in Figure 1.

#### 2.1.2. Data Preprocessing

In order to investigate the effects of different lighting conditions on open-set individual identification, this study selected video data files from barns under conditions of natural daylight and nighttime artificial lighting to acquire images under both natural sunlight and artificial light. Given the high similarity between adjacent frames in video files, the direct frame-by-frame extraction of images could lead to data redundancy, overfitting of the final model, and a limited generalization capability. Therefore, this study processed the video files as follows: The first step was to use FFMPEG software (3.2.19) to perform frame extraction on all collected video files. Specifically, one frame was extracted every ten frames, reducing data redundancy while retaining sufficient image data for a subsequent analysis.

In the second step, to enhance the diversity of the dataset and reduce redundancy, we employed the Structural Similarity Index (SSIM) algorithm [28] for similarity analysis of the extracted images, ultimately setting the SSIM threshold at 0.78. This decision was based on recommended values from similar contexts in previous literature [29] as well as preliminary observational experiments. Comparing datasets generated with various adjacent SSIM values, we noted that when the threshold is set at 0.78, the images in the dataset exhibit sufficient variability to support effective feature learning while maintaining a reasonable volume of data. This threshold ensures that the images removed are primarily those that are highly similar duplicates, thereby optimizing the quality and utility of the training dataset.

In the third step, this study utilized ImageGlass (version V8.8.3.28) software for image cropping and saving. The cropping guidelines were as follows: (1) Only the complete cows in the image were saved as separate images. (2) The boundaries of the cropped individual images were closely aligned with the target. (3) Ensure that the number of images per individual is no less than 40. This approach ensured that the images of each individual in the dataset were sufficiently diverse to support subsequent model training and accurate identification.

In the fourth step, a second dataset check was manually performed. It was verified again whether images of the same cow were assigned to different folders or images of different cows were assigned to the same folder. This process ensured the accuracy and consistency of the dataset. After the selection, elimination, and merging steps above, the final dataset selected comprised complete overhead images of 70 cows.

#### 2.1.3. Dataset Construction

The sizes of the training, validation, and test sets of the final dataset are shown in Table 1. Eight randomly selected cows were used as the test set to achieve open-set individual cow identification. In comparison, the overhead images of the other 62 cows were randomly divided in an 8:2 ratio to construct the training and validation sets. The training set includes 7069 images of 62 cows, the validation set includes 1801 images of 62 cows, and the test set includes 1104 images of 8 cows. The final test set consists of positive and negative sample pairs, with two images randomly drawn from the same cow in the test set as a positive pair, totaling 273 randomly drawn positive image pairs. One image is randomly drawn from different cows to form a negative pair, with 273 negative image pairs randomly drawn to ensure an equal number of positive and negative sample pairs.

**Figure 2:**
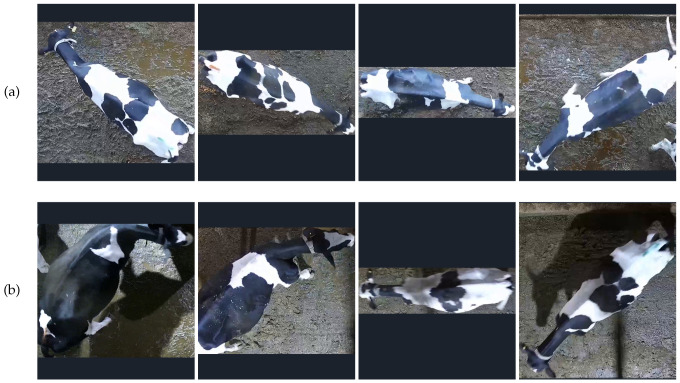
The comparison of images under “natural light” and “artificial light”. (**a**) Image under “natural light”, (**b**) Image under “artificial light”.

**Figure 3:**
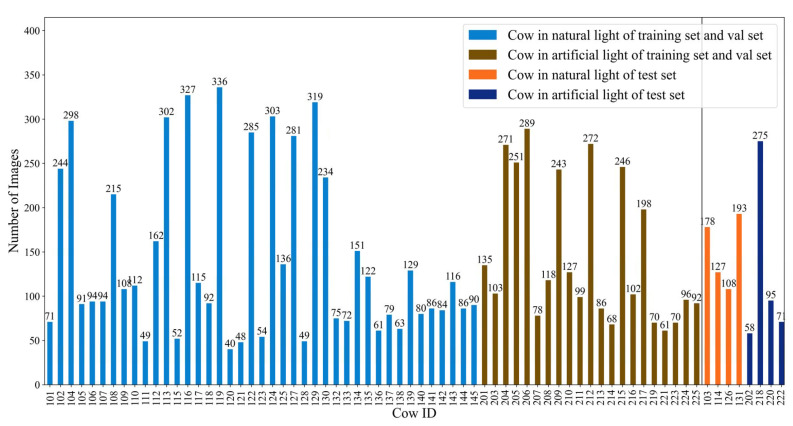
Dataset distribution.

The image samples collected in this study are primarily categorized into two types: natural light and artificial light conditions. Natural light samples were collected outdoors between 6:00 a.m. and 6:30 p.m., depending on external natural lighting. Artificial light samples were gathered indoors under barn lighting conditions from 8:00 p.m. to 10:30 p.m. The comparison of image samples under these two conditions is shown in Figure 2. From Figure 2, it can be observed that images captured under natural light conditions are characterized by high clarity, natural colors, and no shadows, whereas under artificial lighting, the images are generally darker with noticeable shadows, some of which make it difficult to distinguish between shadowed areas and parts of the cattle’s body. Figure 3 details the sample sizes for each cow under these two lighting conditions.

### 2.2. Overview of the Proposed Framework

**Figure 4:**
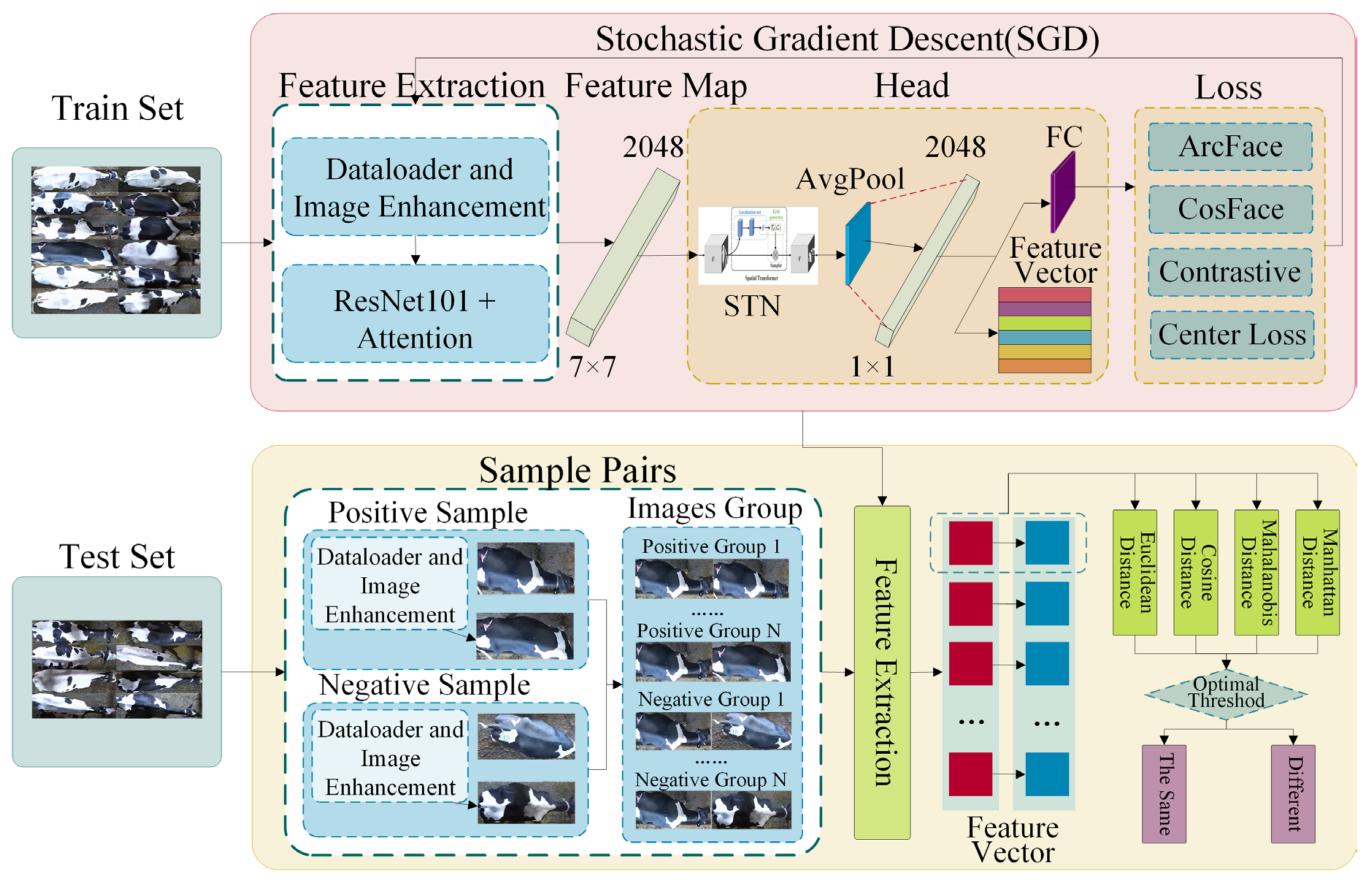
Framework.

The overall workflow of the cow individual identification framework in this study is shown in Figure 4. The entire framework consists of two parts: the feature extraction network and the open-set identification module. This study’s framework introduces an individual identification method for cow overhead views by combining three types of attention mechanisms, four loss functions, and four measurement metrics to explore the open-set identification of cows from a non-contact overhead perspective. The specific process is as follows: First, a feature extraction network for cow overhead views was designed, performing horizontal rotation and random augmentation on the training set data during the data-loading stage to improve the consistency of individual orientations and simulate incomplete individuals and camera soiling in actual production environments. Subsequently, the feature extraction network’s feature maps output undergoes a spatial feature alignment transformation through the STN network, followed by global average pooling to generate feature vectors. Then, after horizontal rotation and random enhancement, positive and negative sample pairs from the test set are input into the feature extraction network to obtain corresponding pairs of feature maps. Finally, metric learning methods are used to measure the similarity of sample pairs, and the best threshold is obtained using a ten-fold cross-validation method, achieving open-set individual identification of cows from an overhead perspective.

### 2.3. Dataloader and Image Enhancement

Since the original data were collected in a production setting, the bodies of the dairy cows could be oriented at various angles, as shown in Figure 2. To minimize the impact of posture changes and different orientations on model recognition efficiency and to enhance the model’s ability to recognize and match features effectively, this study determined whether to rotate images based on their aspect ratio during model training data loading. If the image aspect ratio is greater than or equal to 1, the image is not rotated; if the aspect ratio is less than 1, the image is uniformly rotated clockwise.

Let the width and height of an image be *w* and *h*, respectively; the aspect ratio AspectRatio can be defined as AspectRatio=w/h. The rule to determine whether to rotate the image based on the aspect ratio can be expressed by Equation (1):

(1) $$Rotation=\left\{\begin{array}{cc} 0^{\circ } & \;\mathrm{if}\;\mathrm{Aspect}\;\mathrm{Ratio}\;\geq 1\\ 90^{\circ } & \;\mathrm{if}\;\mathrm{Aspect}\;\mathrm{Ratio}\;<1 \end{array}\right.$$

Here, $0^{\circ }$ means no rotation, while $90^{\circ }$ indicates a clockwise rotation.

**Figure 5:**
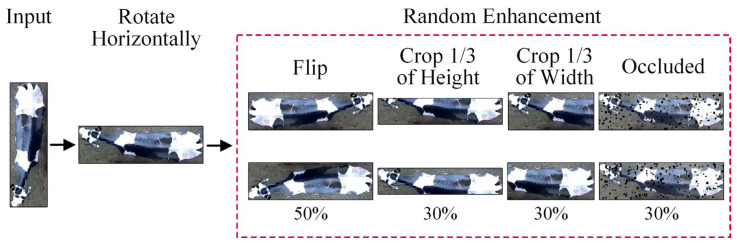
Dataloader and image enhancement.

To better simulate actual production environments and enhance the model’s generalization capabilities, we use the Albumentations library to augment images during model training data loading randomly. This includes a 50% chance of horizontal or vertical flipping, a 30% chance of randomly cropping one-third of the image width from a side, a 30% chance of randomly cropping one-third of the image height from a side, and a 30% chance of small target occlusions occurring (with a maximum height of 18, minimum height of 10, maximum width of 18, and minimum width of 10). The cropping and random occlusion strategies employed in this study aim to simulate visual obstacles that dairy cows may encounter in actual production environments, such as partial occlusions and diverse orientations. The design of these strategies is based on research into visual system preprocessing [30]. Introducing these common real-world issues during training can enhance the model’s adaptability and robustness in complex settings, as shown in Figure 5.

### 2.4. Feature Extraction Network

**Figure 6:**
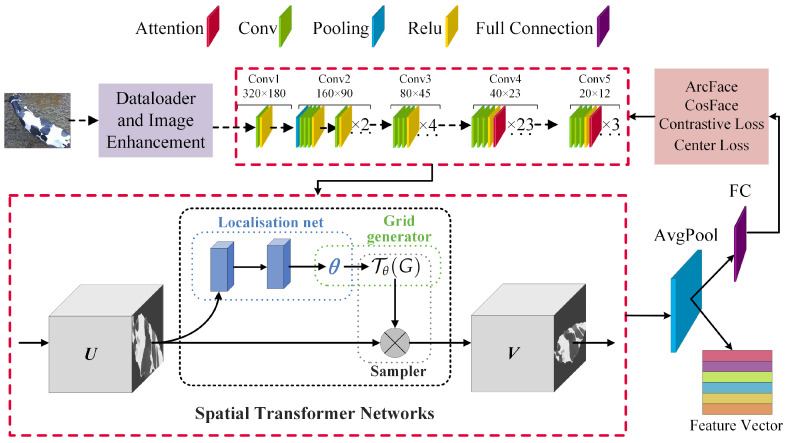
Feature extraction network. Feature extraction network architecture comprising four main components: the backbone network, the attention mechanism, the Spatial Transformer Network (STN) for deep feature extraction, and the loss function. The process includes image augmentation through the data loader and image enhancement module, feature mapping via ResNet101 with an attention mechanism, and feature vector production through STN and average pooling.

Extracting distinguishing features for individual cows from an overhead perspective is a relatively complex task, primarily due to posture differences, orientation, occlusions, background, and changes in lighting. These variability factors demand that the chosen feature extraction network possesses high adaptability and robustness, enabling the effective extraction of individual features from complex backgrounds. The construction of the feature extraction network primarily consists of four parts: the backbone network, attention mechanism, spatial transformation module for deep feature extraction, and the loss function. The feature extraction network architecture is shown in Figure 6.

#### 2.4.1. Backbone Network with Integrated Attention Mechanism

In the study of overhead perspective-based individual cow identification, choosing the appropriate feature extraction network is crucial as it determines the model’s recognition efficiency for the current dataset. The Deep Residual Network (ResNet) is currently the most commonly used feature extraction network and is employed to address the issue of vanishing gradients in deep neural networks. It incorporates residual blocks with skip connections or shortcuts in each block, allowing gradients to flow directly through these connections to enhance the network’s depth. ResNet and its variants are widely used in individual animal recognition [21,24,25] and are considered adequate. ResNet101 is a variant of the ResNet [31] architecture that contains 101 layers. Compared to shallower ResNet variants (such as ResNet50 and ResNet18), ResNet101 enhances the model’s feature-learning capabilities by increasing the number of layers, enabling it to capture more complex features and thus perform better in various visual recognition tasks.

Attention mechanisms are a crucial means of enhancing model feature extraction capabilities and have been proven effective in the field of individual animal recognition [16,25]. Given the diverse orientation distributions and complex lighting conditions in our dataset, we selected seven common attention mechanisms for efficacy screening. These include the BAM (Bottleneck Attention Module) [32], CBAM (Convolutional Block Attention Module) [33], ECA (Efficient Channel Attention) [34], ParNet (Parallel Network) [35], SEA (Squeeze and Excitation Attention) [36], SimAM (Simplified Attention Module) [37], and SK (Selective Kernel Networks) [38].

The CBAM mechanism effectively enhances feature expression by sequentially combining spatial and channel attention. For the individual recognition of dairy cows, spatial attention helps the model focus on the most critical parts of the image, such as distinct body regions. In contrast, channel attention enables the model to highlight feature channels most relevant to the recognition task. The incorporation of CBAM significantly improves the model’s adaptability to spatial locations and feature channels, thereby enhancing the accuracy of the individual recognition of dairy cows in complex backgrounds and various postures. SimAM introduces a simplified attention mechanism designed to provide effective adaptive feature tuning without increasing computational complexity. This mechanism is particularly important for processing large-scale deep image data and optimizing feature representation without significantly increasing the computational load. The ParNet attention module reduces network depth by using parallel subnetworks instead of traditional layer-by-layer stacking while maintaining high performance. It uses a Skip Squeeze and Excitation (SSE) layer based on the Squeeze and Excitation (SE) design, enhancing the network’s receptive field through skip connections and a single fully connected layer without increasing the network depth.

The effectiveness of attention mechanisms is closely related to their placement within the model [39]. In deep neural networks, features at different layers have varying levels of semantic complexity and abstraction. Stages 3 and 4 of ResNet101 are advanced stages responsible for extracting complex abstract features. Adding attention mechanisms at these levels allows for more precise recalibration and optimization of these high-order, semantically rich features. This study specifically opts to insert attention modules at the end of each residual block in Stages 3 and 4 as these layers are deeper within the network and capable of handling more complex background information and varied dairy cow postures. With the inclusion of attention modules, the model can more effectively focus on key features and suppress irrelevant background noise. This strategy is particularly important for processing overhead images of dairy cows as these images often contain complex backgrounds and various occlusion conditions. Through this approach, we are able to significantly improve the model’s accuracy in recognizing individual dairy cows in complex environments, especially under occlusion and varying environmental conditions.

#### 2.4.2. Spatial Transformation Depth Feature Extraction Module

From an overhead perspective of cows, there is a diverse orientation distribution among individuals. Although preliminary angle adjustments were made through aspect ratio determination in the data-loading phase of the experiment, a significant number of individuals with reversed positions and horizontal angle deviations remain. Traditional convolutional neural networks have certain limitations in handling such geometric deformations, struggling to adjust image features to enhance recognition performance adaptively.

The Spatial Transformer Network (STN) is a trainable module designed to enable neural networks to learn to perform spatial transformations of images, thereby enhancing the model’s adaptability to geometric deformations. The STN achieves its functionality through three main components: the Localization network, Grid generator, and Sampler. The Localization network predicts the spatial transformation parameters; the Grid generator creates a sampling grid based on these parameters; and the Sampler then uses this grid to sample from the input image, producing the transformed output image. This process allows the network to perform image correction automatically without manual intervention.

The function of the Localization network is to predict the spatial transformation parameters θ. It is a standard feedforward neural network that takes the input image *I* and outputs the transformation parameters θ, which are then used to generate the Grid generator. The Localization network can be represented by Equation (2):

(2) $$\theta =f_{loc}\left(I\right)$$

Here, $f_{loc}$ denotes the Localization network, *I* is the input image, and θ is the transformation parameters.

Based on the transformation parameters θ provided by the Localization network, the Grid generator generates the corresponding sampling grid. This sampling grid defines the new position of each pixel in the input image. For each output pixel position $\left(x_{i}^{t},y_{i}^{t}\right)$, its corresponding position in the input image $\left(x_{i}^{s},y_{i}^{s}\right)$ is calculated through the affine transformation defined by Equation (3). Here, $T_{\theta }$ is the affine transformation matrix defined by the parameters θ:

(3) $$\left(\begin{array}{c} x_{i}^{s}\\ y_{i}^{s} \end{array}\right)=T_{\theta }\left(\begin{array}{c} x_{i}^{t}\\ y_{i}^{t}\\ 1 \end{array}\right)=\left(\begin{array}{ccc} \theta_{11} & \theta_{12} & \theta_{13}\\ \theta_{21} & \theta_{22} & \theta_{23} \end{array}\right)\left(\begin{array}{c} x_{i}^{t}\\ y_{i}^{t}\\ 1 \end{array}\right)$$

$T_{\theta }$ is the affine transformation matrix defined by the parameters θ.

The Sampler is responsible for sampling from the input image *I* according to the sampling grid produced by the Grid generator, generating the transformed output image $I^{\prime }$. This process involves sampling each new position $\left(x_{i}^{s},y_{i}^{s}\right)$ from the input image to construct the transformed image, using bilinear interpolation for the sampling process.

In research on overhead perspective-based individual cow identification, selecting an appropriate feature extraction network is crucial as it determines the final recognition efficiency for the current dataset. We selected well-known feature extraction networks, including MobileNet V2, ResNet101, ResNeSt101, and Vision Transformer (ViT), as baselines. These networks, encompassing lightweight, residual, and Transformer visual networks and their variants, have been proven effective in the field of individual animal identification [17,21]. In this study, we focus on the model’s performance in processing images of cows from an overhead perspective, characterized by complex backgrounds and variable postures.

#### 2.4.3. Loss Function

This paper integrates and explores four types of loss functions to enhance the performance of the recognition framework: ArcFace loss [40], CosFace loss [41], Contrastive Loss [42], and Center Loss [43]. These loss functions are applied to the model to optimize the distribution of the feature space, aiming to explore the enhancement effects of different loss functions on the model’s recognition capabilities, preparing for open-set recognition based on metric learning. The selection of these loss functions is based on the following considerations: ArcFace loss significantly enhances the distinguishability of individual features by increasing inter-class separation and compressing intra-class distances, a feature crucial for open-set recognition based on distance metrics. CosFace loss directly introduces Cosine margins in the feature space, optimizing the distribution of features between and within classes, which is particularly effective for processing dairy cow images with complex backgrounds and pose variations. Contrastive Loss enhances the model’s discriminative power by ensuring that the distances between samples of the same category are smaller than those between samples of different categories. This is particularly useful for reducing misclassification, especially in open-set environments with a diverse array of sample categories. Center Loss is used to minimize the distance between features of the same class and their class center, effectively enhancing intra-class compactness. This helps the model to stably recognize individual cows under varied viewing angles and occlusion conditions.

ArcFace loss aims to optimize the feature space by enhancing the compactness within classes and separability between classes with the calculation method provided in Equation (4):

(4) $$L_{ArcFace}=-\frac{1}{N}\sum_{i=1}^{N}\log\frac{e^{s\cdot \cos(\theta_{y_{i}}+m)}}{e^{s\cdot \cos(\theta_{y_{i}}+m)}+\sum_{j=1,j\neq y_{i}}^{C}e^{s\cdot \cos(\theta_{j})}}$$

Herein, *N* represents the number of samples in a batch, ensuring that the loss value is independent of the batch size; *s* is a scaling parameter used to adjust the magnitude of the feature vectors; $\theta_{y_{i}}$ denotes the angle between the sample *i* and its corresponding class center; *m* is a preset margin angle, used to enhance separability between classes; and *C* represents the total number of categories.

CosFace loss enhances the separability between classes by directly introducing a Cosine margin into the feature space with the calculation method provided in Equation (5):

(5) $$L_{CosFace}=-\frac{1}{N}\sum_{i=1}^{N}\log\frac{e^{s\cdot (\cos\left(\theta_{y_{i}}\right)-m)}}{e^{s\cdot (\cos\left(\theta_{y_{i}}\right)-m)}+\sum_{j=1,j\neq y_{i}}^{C}e^{s\cdot \cos(\theta_{j})}}$$

The meanings of the variables here are the same as in the ArcFace loss, but the CosFace loss directly subtracts the margin *m* from the Cosine values, optimizing the calculation method.

Contrastive Loss is used to ensure that samples of the same class are closer in feature space while samples of different classes are further apart, with the calculation method provided in Equation (6):

(6) $$L_{Contrastive}=\frac{1}{2N}\sum_{i=1}^{N}\left(1-y_{ij}\right)\cdot ∥f_{i}-f_{j}{∥}^{2}+y_{ij}\cdot \max(0,m-∥f_{i}-f_{j}{∥)}^{2}$$

Here, $y_{ij}$ is a label indicator function, where 0 indicates that samples *i* and *j* belong to the same class and 1 otherwise; $f_{i}$ and $f_{j}$ are the feature vectors of samples *i* and *j*, respectively; and *m* sets the minimum boundary for inter-class distance.

Center Loss aims to minimize the distance between the features of samples of the same class and their class center, as shown in Equation (7):

(7) $$L_{Center}=\frac{1}{2}\sum_{i=1}^{N}{∥f_{i}-c_{y_{i}}∥}^{2}$$

Here, $f_{i}$ refers to the feature vector of sample *i* and $c_{y_{i}}$ represents the feature center of the class to which sample *i* belongs, i.e., the average feature of all samples in that class.

### 2.5. Open-Set Recognition Module

To accurately assess the performance of the open-set recognition system, this study employed a sample-pair-based testing strategy, with the method for generating test set sample pairs detailed in Section 2.1.3. The open-set recognition module involves the random extraction of positive and negative sample pairs, exploration of the application of various distance metrics, and threshold selection based on ten-fold cross-validation. These strategies aim to simulate various scenarios in open-set recognition while optimizing the recognition module’s final performance.

After feature extraction, to understand and determine the similarity of feature vectors in the feature space from different perspectives, this paper explores four distance metrics to determine whether two feature vectors come from the same cow individual based on their similarity. The distance metrics specifically include the Euclidean distance, Cosine distance, Mahalanobis distance, and Manhattan distance. The thresholds for each distance metric are denoted by $d_{et}$, $d_{ct}$, $d_{maht}$, and $d_{mant}$, with their value ranges being $d_{et}\in \left[0,15\right]$, $d_{ct}\in \left[0,1\right]$, $d_{maht}\in \left[0,5\right]$, and $d_{mant}\in \left[0,100\right]$, respectively.

Using the ten-fold cross-validation method and verifying the framework algorithm’s feasibility, this study also aims to find the most appropriate thresholds among these distance metrics to achieve optimal recognition accuracy. In the ten-fold cross-validation process, the test set is divided into ten equal parts, with nine parts selected for training and the remaining part used for testing in each cycle, ensuring that each part can be used as the test set. The top-1 accuracy is used as the standard for evaluating the model, and an exhaustive search for each distance metric determines the best accuracy threshold. The search step size for the Euclidean and Mahalanobis distances is 0.01, for the Cosine distance is 0.0001, and for the Manhattan distance is 0.1. Finally, the optimal thresholds for each distance metric are determined by the weighted average of the accuracy of each test set round, with the collective optimal threshold calculation method shown in Equation (8):

(8) $$d_{\mathrm{opt}}=\frac{\sum_{i=1}^{10}Acc_{i}\times d_{i}}{\sum_{i=1}^{10}Acc_{i}}$$

Herein, $d_{\mathrm{opt}}$ represents the optimal threshold, which is determined by the weighted average of the accuracy rates from each test set in ten-fold cross-validation, aimed at comprehensively reflecting the unified threshold that best represents performance across all cross-validation cycles. $Acc_{i}$ denotes the accuracy on the test set during the *i*th iteration of cross-validation, used to assess the model’s performance under that particular cross-validation iteration. $d_{i}$ represents the best threshold obtained during the *i*th iteration of cross-validation. This value is achieved through exhaustive search or other optimization methods in a specific cross-validation cycle, aiming to maximize model performance (e.g., accuracy). $\sum_{i=1}^{10}Acc_{i}$ represents the sum of accuracies from all test sets across ten cross-validation iterations, serving as the denominator in the weighted average calculation.

### 2.6. Evaluation Indicators

To comprehensively evaluate the performance of the proposed model on the individual cow identification task, this study utilized top-1 and top-5 accuracy as the main evaluation metrics. These metrics intuitively reflect the model’s accuracy and are commonly used standard assessment methods in the fields of image classification and individual identification.

The top-1 accuracy reflects the accuracy of the model in predicting the most likely category; a high value indicates excellent performance in predicting the most probable category. The calculation method is shown in Equation (9):

(9) $$\mathrm{Top}\text{-}1\;\mathrm{Accuracy}=\frac{TP}{TP+FN}$$

Herein, TP represents True Positives, the number of samples correctly predicted as positive by the model and FN stands for False Negatives, the number of samples incorrectly predicted as unfavorable by the model.

The top-5 accuracy assesses whether the model’s predictions for the top five most likely categories include the true category. This metric is particularly applicable to recognition tasks with many categories and high similarity. It reflects the model’s tolerance when there are more options. The calculation method is shown in Equation (10):

(10) $$\mathrm{Top}\text{-}5\;\mathrm{Accuracy}=\frac{\mathrm{Number}\;\mathrm{of}\;T5P}{\mathrm{Total}\;\mathrm{number}\;\mathrm{of}\;\mathrm{predictions}}$$

Herein, “Number of T5P” refers to the number of samples for which the actual label is among the top five categories predicted by the model, and “Total number of predictions” refers to the total number of samples in the test set.

By considering both the top-1 and top-5 metrics, this study comprehensively evaluates the model’s accuracy and generalizability in the task of individual cow identification, thereby verifying the model’s effectiveness and reliability in practical application scenarios.

## 3. Results

### 3.1. Experimental Setup and Parameters

The platform used for this experiment is a Linux server equipped with the Ubuntu Server 22.04 operating system. The server’s hardware configuration includes two Intel(R) Xeon(R) Gold 6139M CPUs @ 2.30 GHz (Intel, Santa Clara, CA, USA), 128 GB of RAM, and 8 NVIDIA GeForce RTX 3090 graphics cards (NVIDIA, Santa Clara, CA, USA). On the software side, the system environment comprises Python 3.10.11; CUDA 11.7; and the deep learning frameworks Pytorch 2.0.1, MMEngine 0.10.3, and MMPretrain 1.2.0. As shown in Table 2, The learning algorithm in this study was optimized using stochastic gradient descent, with an initial learning rate set at 0.02, momentum at 0.9, and a weight decay parameter of 0.0001. Considering the limitations of GPU memory, the batch size was set to 96.

### 3.2. Selection of Prototype Networks for Feature Extraction

**Figure 7:**
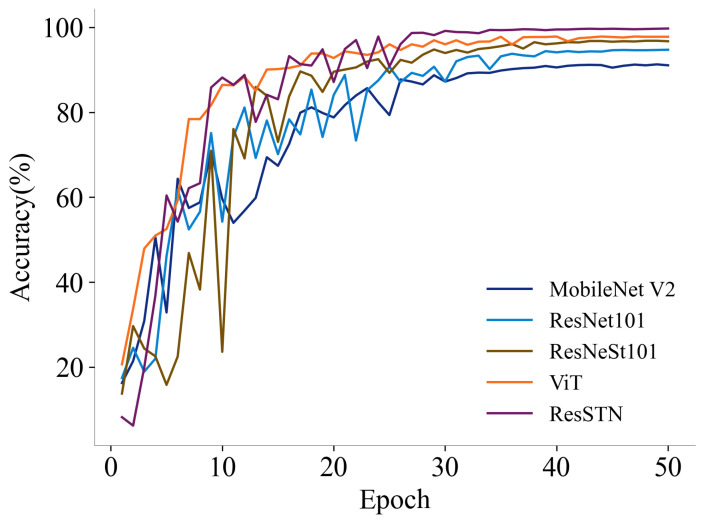
Changes in accuracy during model training.

In this study, we selected MobileNet V2 [44], ResNet101, ResNeSt101 [45], Vision Transformer (ViT) [46], and ResSTN (ours) as the candidate feature extraction networks. We conducted a comprehensive comparison based on their performance on the validation set. As shown in Table 3, in the individual recognition of 62 cows in the validation set, ResSTN performed the best, with a top-1 accuracy of 99.72 and a top-5 accuracy of 99.94. MobileNet V2 performed the worst, yet its top-1 accuracy reached 91.11. ResNet101 was next, with a top-1 accuracy of 94.63. ResNeSt101 and ViT performed slightly worse than ResSTN, with top-1 accuracies of 96.83 and 97.83, respectively. However, the size of the ViT model is about twice that of ResSTN. Considering the top-1 and top-5 accuracies, this study selected ResSTN as the prototype network for cow individual recognition feature extraction. Figure 7 shows the trend of top-1 accuracies for each algorithm during the training process.

### 3.3. Selection of Attention Mechanism for Backbone

As shown in Table 4, in this study, we evaluated the impact of seven common attention mechanisms on the accuracy of the ResSTN model. Different attention mechanisms have varying effects on model accuracy and size. In terms of the top-1 accuracy, CBAM, ParNet, and SimAM exhibited the biggest improvements, reaching 99.85%, 99.91%, and 99.82%, respectively. These mechanisms also performed excellently in top-5 accuracy, scoring 99.96%, 99.97%, and 99.96%, respectively. Compared to the baseline ResSTN model, these three attention mechanisms not only improved the accuracy but also maintained a relatively reasonable model size, particularly CBAM, and SimAM, which had minor increases in the model size. Other attention mechanisms like ECA and SEA, although smaller in model size, resulted in decreased accuracy. Based on these assessments, we selected the three optimal attention mechanisms, CBAM, ParNet, and SimAM, for ablation studies.

### 3.4. Ablation Experiment

This study conducted a series of ablation experiments to investigate the effects of different distance calculation methods, attention mechanisms in feature extraction networks, and loss functions on the task of individual cow identification from an overhead perspective. Initially, the ResNet101 model and ResNet101 models enhanced with three types of attention mechanisms, CBAM, SimAM, and ParNet, were used as feature extraction networks; subsequently, ArcFace, CosFace, Contrastive Loss, and Center Loss were employed as loss functions; finally, the Euclidean distance, Cosine distance, Mahalanobis distance, and Manhattan distance were utilized for distance measurements. These experiments aimed to meticulously analyze the specific impacts of each factor on the model performance, optimize model design, enhance the accuracy and robustness of individual cow identification, and achieve the best performance combination. As shown in Table 5, the ResSTN model, when combined with the SimAM attention mechanism and ArcFace loss function and utilizing the Cosine distance for the distance measurement, achieved the highest accuracy of 94.58%, demonstrating exceptional performance in the task of open-set individual cow identification from an overhead perspective. Furthermore, the ResSTN+CBAM model, in combination with the Contrastive Loss function and the Manhattan distance for the distance measurement, also exhibited a high accuracy of 94.08%, further validating the effectiveness of attention mechanisms in feature extraction and the role of different loss functions in conjunction with various distance measurement methods in enhancing model discriminability.

### 3.5. Comparison of Individual Cow Identification under Different Light and Data Enhancement

This study compared the ResSTN, ResSTN-NE (ResSTN without data augmentation during data loading), and ResNet101-NE (ResNet101 without data augmentation during data loading) as baseline feature extraction models. In conjunction with the SimAM attention mechanism and ArcFace loss function, and using the Cosine distance as the metric, it evaluated the recognition accuracy of whole individuals, randomly cropped individuals, and randomly occluded individuals under natural and artificial lighting conditions. As shown in Table 6, the ResSTN model exhibited the highest recognition accuracy under all test conditions, highlighting the effectiveness of image enhancement techniques, as well as the SimAM attention mechanism and ArcFace loss function. Particularly under natural lighting conditions, the recognition accuracies for whole individuals, randomly cropped individuals, and randomly occluded individuals were 95.23%, 90.85%, and 94.02%, respectively, indicating that recognition performance under natural light generally surpassed that under artificial light. It was also observed that using random data augmentation during the data-loading phase effectively improved the recognition rates for randomly cropped and randomly occluded conditions, with increases of 2.61 and 1.91 percentage points, respectively. Comparing ResSTN-NE and ResNet101-NE, it is evident that the spatial transformations and alignment provided by the STN network significantly enhance the model’s recognition rate by up to 2.98 percentage points.

### 3.6. Comparison with Existing Studies

Table 7 presents the comparative results of this study with other studies. Currently, individual livestock identification is mainly divided into two categories: closed-set identification and open-set identification. In the realm of closed-set identification, existing research primarily focuses on individual identification based on parts of the cow such as the face [17], back [13], side [12,14], and rear [20] using various models including EfficientNet-B1, ResNet50, RetinaFaceNet, VGG-16+SVM, and MobileNet V2. These methods have achieved high recognition accuracy within fixed, predefined sets of individual cows. Research on open-set identification is less common and primarily focuses on the identification of cow bodies and backs. For instance, Andrew et al. [24] achieved a 93.8% recognition accuracy using the RetinaNet (based on ResNet50) model in a test set comprising 46 cows. In contrast, Wang et al. [26] reached an accuracy of 82.93% using the ShuffleNet v2 model on a dataset of 87 cows. Based on these studies, this paper proposes an improved open-set identification framework based on ResSTN. This framework aims to address the challenges posed by diverse orientation distributions and complex lighting conditions in production settings while exploring an overhead perspective cow identification framework based on multiple attention mechanisms, loss functions, and distance metrics. The model proposed in this study ultimately achieved a recognition accuracy of 94.58% under varied orientation distributions and complex lighting conditions, surpassing existing open-set identification models and demonstrating the effectiveness of this method in recognizing individual cows in production scenarios.

## 4. Discussion

According to the results in Table 3, the ResSTN model demonstrates exceptional performance in the individual cow recognition task on the validation set, achieving top-1 and top-5 accuracies of 99.72% and 99.94%, respectively. Although the size of the ViT model is approximately twice that of ResSTN, its performance does not surpass that of ResSTN, indicating that increasing the model size does not always linearly improve performance in individual recognition. Conversely, ResSTN achieves higher recognition accuracy while maintaining a more petite model size. This suggests that the STN network within the ResSTN structure reduces the impact of individual posture and angle variations on the final model performance, decreasing the complexity of transformations and deformations that ResNet needs to handle, thereby improving the quality of feature extraction. It also indirectly reflects the importance of data alignment for individual recognition, consistent with the research findings of Wang et al. (2023) [26].

According to the results shown in Table 4, CBAM and SimAM significantly improved the top-1 and top-5 accuracy of the model with only a slight increase in the model size. In contrast, although ParNet increased in size, its parallel structure enabled it to still exhibit an outstanding performance in recognition tasks. These results indicate that in practical applications, choosing the appropriate attention mechanism can effectively enhance model performance without significantly increasing the consumption of computational resources. Additionally, other attention mechanisms such as ECA and SEA, while advantageous in terms of model size, did not improve accuracy and even resulted in performance degradation. This may be due to these mechanisms’ inability to adequately capture features beneficial for improving classification accuracy in this research context.

Table 5 shows that although all model variants demonstrate a high recognition accuracy, this study finds that the Cosine distance achieves a good performance across various configurations, especially when combined with the SimAM attention mechanism. Furthermore, the results of ablation studies also reveal the significant impact of loss function selection on model performance. For instance, the ArcFace loss function achieves high accuracy across various attention mechanism configurations, indicating its advantage in promoting the model to learn more discriminative features.

To mitigate the negative impact of sample class imbalance on recognition accuracy and to evaluate the best threshold, this study employed a ten-fold cross-validation method to estimate the model’s average accuracy. Specifically, the test sample pairs are divided into ten parts, with nine parts selected in each round as the basis for determining the optimal threshold, which is then used to evaluate the accuracy of the remaining part. This process is repeated ten times, with a different part selected as the test set each time, thus obtaining ten accuracy values. The weighted average of these accuracies is considered the model’s average performance across the entire test set.

According to Table 8, the model exhibited an average accuracy of 94.58% when using the Cosine distance as the distance metric, the highest among the four distance measurement algorithms. Furthermore, both the Euclidean distance and Mahalanobis distance showed the same average accuracy of 92.90%, while the Manhattan distance had an average accuracy of 93.46%. The accuracy results from the ten-fold cross-validation are consistent with the ablation experiment outcomes. Simultaneously, the optimal thresholds for the Euclidean, Cosine, Mahalanobis, and Manhattan distance were identified as 2.45, 0.0047, 4.24, and 72.2, respectively. Regardless of the average or highest accuracy, the Cosine distance outperformed the other distances, indicating its suitability for individual identification tasks, in agreement with the findings of Wang et al. (2023) [25]. The excellent performance of the Cosine distance in this study may be attributed to its measurement approach, which evaluates similarity by calculating the Cosine of the angle between two feature vectors, a method insensitive to vector lengths and more focused on directional similarity.

As shown in Table 6, the recognition accuracy of whole-body images under natural lighting surpassed that under artificial lighting, reaching 95.23% in natural light compared to 94.86% under artificial light, indicating that natural lighting provides better lighting consistency and feature clarity for model recognition. The accuracy difference between natural and artificial lighting conditions was minimal for randomly cropped individuals, likely because cropping reduced the impact of lighting changes on overall image features. However, the recognition accuracy of randomly cropped images was lower than that of whole-body images. However, an accuracy of 90.85% remained high, suggesting that the random cropping of images during data loading positively impacts the model’s ability to identify non-complete individuals.

Moreover, comparing the ResSTN-NE and ResNet101-NE models, which did not use data augmentation strategies, further confirmed the significant role of data augmentation in enhancing model robustness and adaptability. The recognition performance under natural lighting conditions was generally superior to that under artificial lighting, emphasizing the importance of considering lighting conditions in practical applications. Data augmentation techniques for random cropping and occlusion significantly improved recognition accuracy, indicating that data augmentation enhanced the model’s ability to utilize local image information and process occluded and incomplete images. This finding aligns with the current deep learning field’s general recognition of data augmentation techniques. Simulating various disturbances that might occur during the training process improves the model’s generalization ability and robustness. Comparing the performance of the ResSTN-NE and ResNet101-NE models demonstrated the positive impact of spatial transformation and alignment capabilities provided by the STN network on enhancing model recognition rates, with an average accuracy improvement of 2.98 percentage points. Especially when processing whole-body, randomly cropped, and randomly occluded individuals under natural light, the high accuracy displayed by the ResSTN model reaffirmed the effectiveness of the STN structure in handling image geometric transformations and alignments, which is crucial for improving the performance of vision-based individual identification systems.

**Figure 8:**
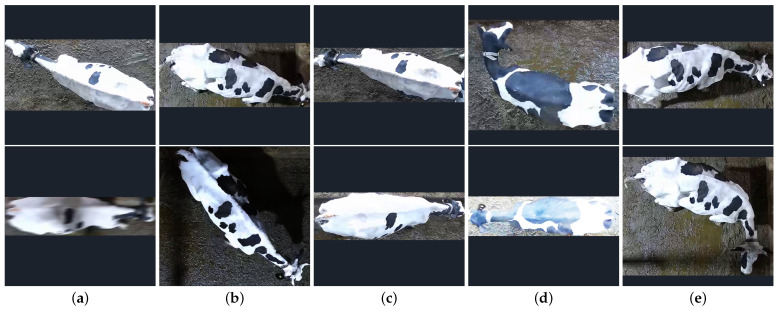
Examples of recognition errors. (**a**) Image distortion caused by rapid movement, (**b**) low brightness under lighting conditions, (**c**) insufficient individual cow features + feature loss due to random cropping, (**d**) camera overexposure, (**e**) additional black patches on the cow’s back due to light shadows.

This study conducted an in-depth analysis of individual cow recognition from an overhead perspective. Despite using the advanced ResSTN model, combining attention mechanisms and various distance metrics algorithms, the experimental results show a recognition accuracy of up to 94.58%. However, recognition errors still occur under certain conditions. Through a case analysis of misrecognition, this study found that the main reasons for recognition errors can be categorized into five types, as shown in Figure 8. Figure 8a shows a case of image distortion caused by the rapid movement of cows. When cows move quickly, the images captured by the camera may become blurred or distorted, making the individual cow’s feature information less distinct and affecting the model’s recognition accuracy. Significantly, when the movement speed exceeds the camera’s capturing ability, the degree of image distortion becomes more severe. Figure 8b shows that lighting conditions also significantly affect model performance. Under low-light conditions, the black features of cows blend with light shadows, increasing the likelihood of recognition errors. Figure 8c shows that the individual cow has too few distinguishable features, with only sporadic black spots on the right side and the rest of the area white. Random cropping or occlusion for data augmentation to improve the model’s generalization capability may result in the loss of some or all recognizable features, leading to insufficient feature information for the cow individual and making it difficult for the model to extract adequate recognition information from the image. As shown in Figure 8d, overexposure of the camera leads to the loss of details in the image, especially when the background color is similar to the cow’s fur. Overexposure can make the cow’s contour and features less distinct, affecting the model’s ability to recognize. As shown in Figure 8e, shadows may form additional black or gray patches on the cow’s back under certain lighting conditions. The model may misidentify these patches as one of the cow’s features, leading to recognition errors. The risk of misidentification may be greater, especially when the shadow’s shape is similar in brightness to the cow’s natural markings.

While this study has made certain progress in open-set individual cow recognition, limitations may affect the model’s general applicability and final performance.

First, the training, validation, and testing of the model in this study were primarily based on a relatively closed and controllable dataset. Therefore, the model may face challenges when dealing with more extensive and diverse individual cow characteristics, especially when there are few discernible features per individual cow. Although data augmentation techniques can somewhat enhance the model’s generalization capabilities, future research will need to train and validate more diverse datasets to improve the model’s practicality and robustness further.

Secondly, this study directly referenced existing research on the placement of attention mechanisms within the feature extraction network without conducting more in-depth ablation analyses. It is possible that the current method of adding attention mechanisms is not optimal. Future work could explore the impact of different placements of attention mechanisms under an overhead perspective of cows on the ability to extract open-set recognition features.

Thirdly, the parameter count of the ResNet-based feature extraction network models is relatively large, especially after adding mechanisms such as attention. The larger model parameter count increases the implementation cost in production, posing a significant challenge for the model’s application and promotion. Future work could explore lightweight models suitable for end-point deployment and lightweight open-set recognition systems.

Lastly, this study has partially addressed the challenges of partial cow visibility and diverse orientation distribution under an overhead perspective for individual identification. However, it has not fully considered all challenges in actual production environments. For example, the impact of temporary artificial markings on the cow’s back, severe back contamination, skin diseases, and feature changes due to individual growth on the accuracy of identification. Future research needs to study the impact of various and multiple factors in complex production environments on model performance and corresponding optimization strategies, thereby developing more robust and suitable individual cow identification models for complex production environments.

Future research needs to delve deeper into the specific impact of these factors on model performance, thereby identifying more robust and applicable cow individual recognition models for complex production environments.

## 5. Conclusions

This study constructed an open-set recognition framework that combines “deep feature extraction and metric learning”, effectively enhancing the model’s adaptability to challenges such as the diverse orientation distribution of individual cows, complex lighting conditions, and partial occlusions. This includes designing a new deep learning model, ResSTN, integrating three types of attention mechanisms and four types of loss functions, and exploring the impact of four metric learning methods on recognition performance. Experimental results show that the ResSTN model achieves a recognition accuracy of 94.58% when combined with the SimAM attention mechanism and ArcFace loss function, using the Cosine distance as the distance metric. Under natural lighting conditions, the recognition accuracy for complete cow images reaches 95.23%. This study provides an effective solution for the open-set recognition of individual cows on a technical level and offers rich technical references for future research in related fields. However, transferring the application to natural production environments presents some potential challenges. In future work, we will focus on collecting a broader dataset and optimizing the model architecture to address usability challenges in natural production environments.

## Figures and Tables

**Table 1:** Open-set cow-back-recognition dataset.

Dataset	Number of Cows in Natural Light	Number of Cows in Artificial Light	Total Number of Cows	Total Number of Images
Train dataset	41	21	62	7069
Val dataset	41	21	62	1801
Test dataset	4	4	8	1104
Total	45	25	70	9974

**Table 2:** Experimental parameter setting.

Hyperparameters	Value
Optimizer	SGD
Learning rate	0.02 with scheduler
Momentum	0.9
Weight decay	0.0001
Batch size	96
Max epochs	50
Warm up learning rate scheduler	LinearLR (Epoch 1–5)
Main learning rate scheduler	LinearLR (Epoch 6–50)
Learning rate update	by epoch

**Table 3:** Feature extraction prototype network comparison.

Model	Size (MB)	Accuracy/Top-1 (%)	Accuracy/Top-5 (%)
MobileNet V2	9.32	91.11	93.72
ResNet101	163.51	94.63	96.83
ResNeSt101	98.2	96.83	98.83
Vision Transformer (ViT)	327.78	97.83	99.78
ResSTN (ours)	167.85	99.72	99.94

**Table 4:** Attention mechanism selection.

Model	Size (MB)	Accuracy/Top-1 (%)	Accuracy/Top-5 (%)
ResSTN	167.85	99.72	99.94
ResSTN-BAM	212.34	99.75	99.94
ResSTN-CBAM	186.21	99.85	99.96
ResSTN-ECA	167.87	97.52	98.68
ResSTN-ParNet	1761.6	99.91	99.97
ResSTN-SEA	185.96	97.06	98.57
ResSTN-SimAM	167.84	99.82	99.96
ResSTN-SK	1644.32	97.22	99.28

**Table 5:** Ablation experiment.

Model	Loss	Accuracy (%)			
		Euclidean Distance	Cosine Distance	Mahalanobis Distance	Manhattan Distance
ResSTN	ArcFace	91.77	94.02	89.52	90.08
	CosFace	79.95	79.38	72.06	81.07
	Contrastive Loss	90.08	90.08	79.95	90.08
	Center Loss	77.13	86.14	71.50	74.88
ResSTN + CBAM	ArcFace	83.89	92.33	83.32	83.89
	CosFace	82.76	90.64	71.50	81.64
	Contrastive Loss	92.96	93.52	86.80	94.08
	Center Loss	91.77	92.33	90.08	92.90
ResSTN + SimAM	ArcFace	92.90	**94.58**	92.90	93.46
	CosFace	87.27	83.89	79.95	87.83
	Contrastive Loss	92.90	92.90	84.45	90.64
	Center Loss	82.20	91.77	73.75	79.38
ResSTN + ParNet	ArcFace	85.58	93.46	72.06	74.32
	CosFace	69.25	91.77	64.75	67.56
	Contrastive Loss	68.12	82.76	62.49	65.31
	Center Loss	79.95	83.89	79.95	79.95

**Table 6:** Detection performance on the six test sets.

Evaluation Indicator	Nature Light			Artificial Light		
	**Full-Body**	**Randomly Cropped**	**Randomly Occluded**	**Full-Body**	**Randomly Cropped**	**Randomly Occluded**
ResSTN	95.23%	90.85%	94.02%	94.86%	90.02%	93.87%
ResSTN-NE	94.47%	88.24%	92.11%	93.20%	88.04%	91.61%
ResNet101-NE	91.35%	85.82%	90.25%	89.38%	85.32%	87.66%

**Table 7:** Comparison with existing studies.

Recognition Type	Paper	Year	Objects	Parts	Backbone	Accuracy
Closed set	Hou et al. [20]	2021	195 cows	Rump	MobileNet V2	99.76%
	Xu et al. [17]	2022	90 cows	Face	RetinaFace(MobileNet)	91.3%
	Xiao et al. [13]	2022	48 cows	Back	Mask R-CNN + SVM	98.67%
	Fu et al. [21]	2022	13 cows	Body	ResNet50	98.58%
	Zhang et al. [14]	2023	118 cows	Body	EfficientNet-B1	98.5%
Open set	Andrew et al. [24]	2021	46 cows	Back	RetinaNet (ResNet 50)	93.8%
	Wang et al. [26]	2023	87 cows	Body	ShuffleNet v2	82.93%
	Ours	2024	70 cows	Top-down view	ResSTN%	94.58%

**Table 8:** Ten-fold cross-validation.

Test	Euclidean Distance		Cosine Distance		Mahalanobis Distance		Manhattan Distance	
	**Optimal Threshold**	**Accuracy**	**Optimal Threshold**	**Accuracy**	**Optimal Threshold**	**Accuracy**	**Optimal Threshold**	**Accuracy**
Fold 1	2.45	94.85	0.0047	96.89	4.24	94.85	72.2	93.21
Fold 2	2.45	94.92	0.0047	95.95	4.24	94.92	72.2	93.54
Fold 3	2.45	94.85	0.0047	94.89	4.24	94.85	72.2	93.32
Fold 4	2.45	96.41	0.0047	97.88	4.24	96.41	72.2	96.70
Fold 5	2.45	92.84	0.0047	94.75	4.24	92.84	72.2	94.68
Fold 6	2.45	92.82	0.0047	94.85	4.24	92.82	72.2	93.58
Fold 7	2.45	88.91	0.0047	89.72	4.24	88.91	72.2	90.27
Fold 8	2.45	89.29	0.0047	91.68	4.24	89.29	72.2	91.59
Fold 9	2.45	91.54	0.0047	93.66	4.24	91.54	72.2	93.12
Fold 10	2.45	92.55	0.0047	95.56	4.24	92.55	72.2	94.63
Average								
Accuracy	-	92.90	-	94.58	-	92.90	-	93.46

## Data Availability

The data presented in this study are available from the corresponding author upon reasonable request. The data are not publicly available due to [privacy and confidentiality agreements that protect the commercial interests of the farms involved].

## Abbreviations

The following abbreviations are used in this manuscript:

STN	Spatial Transformer Network
ResNet	Residual Network
NVR	Network Video Recorder
SSIM	Structure Similarity Index Measure
SGD	Stochastic gradient descent
CBAM	Convolutional Block Attention Module
SimAM	Simplified Attention Module
ParNet	Position Attention and Relation Network
TP	True Positive
FN	False Negative
ViT	Vision Transformer
